# Bioinspired Materials for Wound Healing Application: The Potential of Silk Fibroin

**DOI:** 10.3390/ma13153361

**Published:** 2020-07-29

**Authors:** Mauro Pollini, Federica Paladini

**Affiliations:** 1 Department of Engineering for Innovation, University of Salento, Via Monteroni, 73100 Lecce, Italy; 2 Caresilk S.r.l.s., Via Monteroni c/o Technological District DHITECH, 73100 Lecce, Italy

**Keywords:** silk, fibroin, wound healing, antibacterial

## Abstract

Nature is an incredible source of inspiration for scientific research due to the multiple examples of sophisticated structures and architectures which have evolved for billions of years in different environments. Numerous biomaterials have evolved toward high level functions and performances, which can be exploited for designing novel biomedical devices. Naturally derived biopolymers, in particular, offer a wide range of chances to design appropriate substrates for tissue regeneration and wound healing applications. Wound management still represents a challenging field which requires continuous efforts in scientific research for definition of novel approaches to facilitate and promote wound healing and tissue regeneration, particularly where the conventional therapies fail. Moreover, big concerns associated to the risk of wound infections and antibiotic resistance have stimulated the scientific research toward the definition of products with simultaneous regenerative and antimicrobial properties. Among the bioinspired materials for wound healing, this review focuses attention on a protein derived from the silkworm cocoon, namely silk fibroin, which is characterized by incredible biological features and wound healing capability. As demonstrated by the increasing number of publications, today fibroin has received great attention for providing valuable options for fabrication of biomedical devices and products for tissue engineering. In combination with antimicrobial agents, particularly with silver nanoparticles, fibroin also allows the development of products with improved wound healing and antibacterial properties. This review aims at providing the reader with a comprehensive analysis of the most recent findings on silk fibroin, presenting studies and results demonstrating its effective role in wound healing and its great potential for wound healing applications.

## 1. Nature as Source for Scientific Inspiration

Having evolved for billions of years, nature offers a huge variety of materials and structures with different functions and properties [1]. The natural world and its nano- and micro-structured materials have attracted the interest of scientists and have inspired the scientific research towards the definition of synthetic structures that can mimic their features and functions [2]. All natural materials are characterized by some common architectures, and most of them have a composite structure organized into a multilevel hierarchical combination of building blocks with precise patterns and distinctive qualities [1,2,3]. The basic building block of life is represented by the living cell, where multiple organelles, processes and signals are located [4]. In particular, the extracellular matrix (ECM), an intricate network made of different components such as collagen, laminin, fibronectin etc., contains important biochemical and mechanical signals responsible for homeostasis and cell functions [5,6]. By mimicking the natural ECM, novel biomaterials can be developed for tissue engineering and regenerative medicine in order to provide mechanical support and to control the cell behavior [5,6]. Biomaterials for functional tissue repair can be natural or synthetic [5] and different approaches have been developed so far to design biomaterials based on the ECM, ECM-like materials and ECM-synthetic polymer hybrid materials [6]. Although synthetic polymers can be considered more advantageous in terms of reproducibility, in most cases they are inert and do not properly interact with cells [6]. On the other hand, natural biocompatible and biodegradable biopolymers, such as proteins and polysaccharides, can provide the highest degree of biomimicry in reproducing the physicochemical properties of the native ECM, thus providing a versatile platform for biological environments [7]. Moreover, many natural materials are characterized by the capability to change their physicochemical properties in a stimulus-responsive manner, that is inspiring for the development of adaptive artificial materials [8]. Bioinspiration, defined as a “product or process influenced or informed by biology”, can translate a certain biological design into a useful technology [9], arising from natural structures and cell environments, or biological anomalies such as shark skin or the adhesive proteins in marine mussels [10]. Biomimetics, the science of imitating nature, is an extremely exciting field that investigates the biological world and applies its solutions to the science of materials [3]. The bioinspired research aims at developing new materials and providing technological approaches through a comprehensive understanding of the interaction between materials and cells at any length scale [10]. Composition, structure, physicochemical properties and bioactive factors are some examples of sources for naturally-derived and bioinspired materials [11], which can be designed at various scales through imaging, simulation and mathematical modeling tools [12]. Interesting applications of bioinspired and biomimetic nanomedicines have been proposed for treating different diseases through unique designs of structure and function [13]. Among the different applications of bioinspired architectures, bioactive materials mostly based on natural proteins and polysaccharides have been proposed for wound management [7]. Indeed, wound care still represents an increasing public healthcare concern and a big challenge for clinicians due to limited efficacy, high costs and the length of current treatments [14]. Moreover, the increased number of diabetic and ageing populations has increased the number of diseases associated with wounds; any impairment in the complex wound healing process can determine the onset of chronic wounds and the failure of the wound management [7,14]. In this scenario, the definition of novel biomaterials for providing more effective approaches in wound care is urgently required [14]. Regenerative medicine offers many approaches for promoting wound healing, involving the use of growth factors, stem cells and biomaterials, which can be used to repair or modify the wound environment and stimulate the healing process [14]. Evolution has generated numerous biomaterials evolved in many different environments toward high level functions and performances, which can be exploited for designing novel biomedical devices [9]. Naturally derived biopolymers, in particular, offer a wide range of chances to design appropriate substrates for tissue regeneration [7].

## 2. Biomaterials for Wound Healing

The skin is responsible for many biological functions, including thermoregulation, hydration, and synthesis of vitamin D [15]. When the integrity of the skin is impaired, a wound occurs, engaging even muscles, nerves and organs in the case of deep injuries [16]. High morbidity and mortality are associated to major skin injuries [15]. Non-healing chronic wounds represent a serious concern for individuals, healthcare systems and they are a great challenge for doctors [17], particularly in patients with diabetes, who can be affected by limb ulcers with serious consequences, even amputation and death [18,19]. The main goal of all wound management is quick wound healing with restoration of functions and aesthetic appearance [16].

Wound healing is a complex and dynamic process which involves many interactions between cells, secretary factors and ECM matrices [20] in the different exudative, resorptive, proliferative, regenerative phases [16,21]. Indeed, in the exudative phase, when a visible clot is formed, various growth factors are secreted by platelets and, in turn, macrophages and fibroblasts are activated and cytokines are released. The following resorptive phase (inflammation) is controlled by leucocytes and macrophages and by the immune system while, in the proliferative phase, fibroblasts and growth factors are responsible for the formation of the new extracellular matrix and granulation tissue.

Epithelization and collagen synthesis occur in the regenerative phase, followed by remodelling and restoration of functions and processes [16]. Promotion of tissue healing is considered today as a challenging step towards a complete regeneration and, in this regard, the understanding of cell–cell and cell–ECM interactions in both healthy and impaired wound healing is crucial to identify the most appropriate wound management strategies [22]. Diabetes, in particular, negatively affects the highly coordinated events of the wound healing process. A better understanding of the mechanisms associated to wound healing, the wound environment and pathophysiological conditions is necessary for the development of enhanced and targeted wound healing strategies involving different aspects of material science, cellular and molecular biology [18,19].

Better biomaterials for controlled release of signaling molecules, such as growth factors and cytokines, can be engineered to reproduce the natural extracellular matrix and to promote angiogenesis and re-epithelization, thus promoting the wound healing process [19].

Recent progresses in regenerative medicine, nanotechnologies and bioengineering have provided useful platforms to improve knowledge on tissue engineering for the development of effective biomaterials to replace the ECM and restore damaged tissues [20], creating living three dimensional tissues by means of biological substitutes obtained by a combination of scaffolds, cells and signals [23]. Various products have been developed to repair different skin lesions according to the different types of wounds [21], and new active molecules and mechanisms in the healing process have been investigated in biology and pharmaceutical sciences [24]. Bioderived materials, in particular, have demonstrated a significant potential in tissue injury treatment and in improved wound healing, due to their excellent biocompatibility, bioactivity and capability to induce skin tissue repair [17,20]. Biomaterials with wound healing capability have been explored for different applications in wound management, such as for providing a favorable microenvironment for cell growth due to bioresponsive and biomimetic properties, for reducing microbial colonization and for delivery of therapeutic molecules [20,22].

In this scenario, an extremely valuable bioderived material for wound healing application is represented by silk, which is attracting today the great interest of researchers and companies for the interesting biological properties attributed to its proteins: fibroin and sericin [25].

## 3. Silk Fibroin as a Nature Derived Material for Wound Healing Application

A cocoon is a biological composite material with a hierarchical structure made of silk fibers in a sericin matrix [26]. During the metamorphosis from larvae to moth, the domesticated *Bombyx mori* silkworm produces and spins a high amount of silk by using specific glands [27]. Ecologists assess that this structure has evolved over millions of years to optimize the protection of the silkworm pupae from the attack of animals and bacteria and from adverse environmental conditions [26]. Compared with spiders, which also spin silk to swath their prey, the *Bombyx mori* offers the advantage of being able to be reared in captivity. Spider rearing is also more difficult due to the cannibalistic nature of most species and it is less advantageous due to the lower quantities of produced silk [27,28].

Some wild silkworm species, which secrete a silk fibroin with higher cell affinity and adhesion due to the motif arginine-glycine-aspartate (RGD) in the protein chain, also result unsuitable for domestication, thus resulting in them being less indicated for biomaterial applications [29]. Silk represents a valuable biomaterial for various medical and pharmaceutical applications [30] and, beyond its well-known use as a suture material, the intense research and in vivo and in vitro studies on silk have revealed its huge potential for different clinical treatments [31], and for many biomedical applications due to its tunable properties and the possibility for it to be combined with other materials such as proteins, polymers and ceramics for improved properties and functions [30,31]. Silk fibroin (SF), protein derived from the domesticated *Bombyx mori*, has demonstrated interesting features for textiles, drug delivery, imaging, and tissue engineering applications [32]. It is also a Food and Drug Administration (FDA) approved material for some biomedical applications [33] and in tissue engineering, which involves the definition of approaches to repair/replace damaged or non-functional tissues and organs, it represents a valuable natural biopolymer with many advantages such as good biocompatibility and biodegradability, thermal stability and excellent mechanical properties, allowing minimal immune response and good cell adhesion and growth [30]. In silk fibroin, the mechanical properties are predominantly associated to the interactions within its building blocks. Silk fibroin is mainly composed of hydrophobic β-sheet crystallites and hydrophilic amorphous domains. The crystallite domains are composed of heavy-and light-chain polypetides, predominately containing the amino acids glycine (Gly) and alanine (Ala); hydrogen bonds provide a β-sheets anti-parallel arrangement by holding the adjacent chains [30,34]. This multi-domain natural protein has demonstrated superior stretchability and biocompatibility, as well as versatile biodegradability, processability [35] and thermal stability up to approximately ~250 °C [34]. Silk is also a robust structural material with higher resilience against changes in temperature, moisture and pH than other biopolymers [36]. In the form of hydrogel, sponge, film, electrospun nanofiber, silk fibroin (SF) has demonstrated excellent properties as a wound dressing biomaterial, such as maintenance of a moist environment and gas permeability [37,38], improved cell growth, proliferation and migration of different cells lines involved in the different phases of the wound healing process [38,39,40]. One of the main parameters involved in the regulation of the wound healing process is represented by the interaction between the different cells and ECM components and, in these biological mechanisms, silk biomaterials can play a key role for wound healing [41]. Fibroin matrices accelerate cellular adhesion, wound contraction, re-epithelialization, angiogenesis and collagen formation [33].

In both in vitro and in vivo studies on wound healing, SF-based biomaterials have demonstrated good cell adhesion and fibroblast proliferation, with improved neovascularization [42], faster and better tissue healing and complete regeneration in a rat model [43].

**Figure 1:**
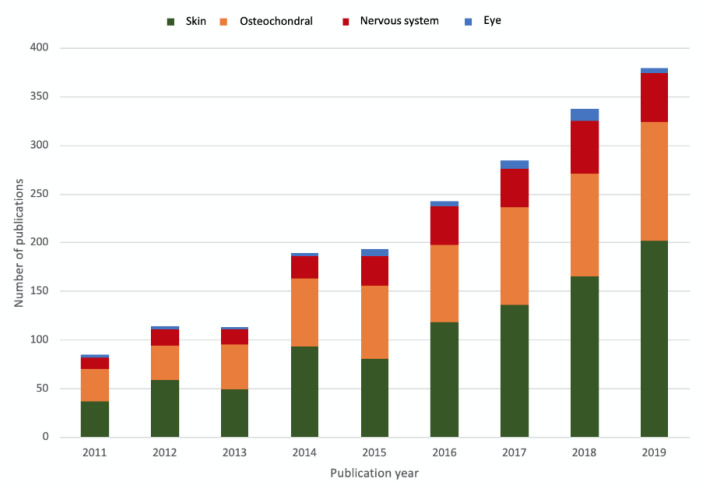
Number of publications per year (source: Scopus), showing the increased interest in the application of silk fibroin for different applications in tissue engineering (skin, osteochondral, nervous system, eye).

On cutaneous wounds generated on the dorsum of New Zealand rabbits, silk fibroin sol-gel films demonstrated better wound healing than standard dressings, and histological analysis also revealed a successful reconstruction of the epidermis. The quantitative evaluation of wound-healing was performed by the authors by measuring untreated and fibroin-treated wound areas. After 10 days of treatment with fibroin biomaterials, the wound size was reduced to about 30%, and further reduced to about 11% after 15 days, while in the control samples it resulted about 52% and 49%, respectively [44]. Sultan et al. have reviewed different studies about the effect of silk fibroin on different cell lines and molecular signaling involved in wound healing. For example, cytokines and growth factors have been recognized as crucial in wound healing, and some studies have demonstrated the role of silk fibroin in suppressing the increased proinflammatory cytokines during the inflammation phase of wound, thus resulting in a protective effect in cells and tissues during the wound healing process. Inactivation of the apoptotic pathway along with stimulated cell migration are other effects also attributed to silk fibroin [45]. To promote functional tissue regeneration, a biomaterial should support and promote biological functions, being properly compliant with the specific needs of the different tissues [46] and, for this purpose, fibroin is a very promising material for tissue engineering. Indeed, as reported in Figure 1, the number of publications devoted to the application of silk fibroin in different fields of tissue engineering has recently increased, thus demonstrating the growing interest of scientific research towards this interesting biomaterial.

Silk fibroin was also demonstrated to have a positive effect in the treatment of hypertrophic scars, which are characterized by an excessive deposition of fibroblast-derived ECM proteins and by persistent inflammation and fibrosis. In that study, fibroin whitened the scar colour and reduced its thickness [47]. The mechanical properties of silk scaffolds can also provide physical stimuli for cell differentiation into endothelial cells and neovascularization without the presence of growth factors [48]. The effect of silk fibroin on cell adhesion, migration and differentiation is related to the scale structure. Single structures inspired by nature have evolved in multi-level structures obtained by novel techniques such as micropatterning and 3D printing, which have exhibited a multifunctional integration with biological systems [49]. Three-dimensional silk biomaterial scaffolds with high compressive strength and interconnected pores suitable for biomedical applications were obtained through salt leaching and gas foaming techniques [50].

**Figure 2:**
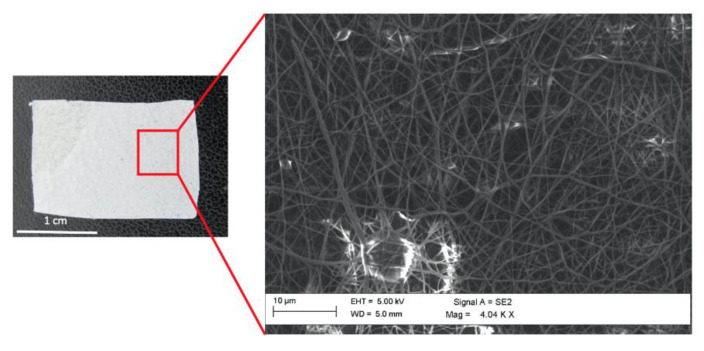
Electrospun fibroin sample (**left**); scanning electron microscopy (SEM) analysis (**right**) showing the fibroin fibers obtained through electrospinning technique.

Other manufacturing methods such as fiber bonding, phase separation, solvent casting etc., have been proposed to fabricate three dimensional porous scaffolds and, more interestingly, 3D printing techniques including rapid prototyping and additive manufacturing have emerged for diverse medical applications for producing scaffolds with controlled pore size, architecture, mechanical and biological properties [51,52]. Major control of the cellular environment can be achieved with 3D bioprinting, which is an advanced technology adopted to create tissues with different cell types and to mimic the three-dimensional geometry and structure of native tissues and organs [53,54,55]. Differently from 3D printing, bioprinting prints cell-laden bioinks [53], enabling the production of scaffolds with a homogeneous distribution of cells throughout a scaffold and mimicking natural-like extracellular matrices and tissues with multiple cell types [56,57]. Proposed for different application areas, such tissue engineering, regenerative medicine research, transplantation, clinics etc., [58], bioprinting technologies have demonstrated some advantages i.e., precise control and repeatability, but many aspects still remain challenging for building complex tissues and structures [57,59]. For wound healing applications, bioprinted skin substitutes offer a promising approach in skin bioengineering for developing fully functional skin constructs [60,61]. A suitable biopolymer mixed with various cells, such as keratinocytes, fibroblasts and melanocytes cells, is used to form bioink and fed to the bioprinting system [60]. In this scenario, crucial aspects for the development of new therapeutic strategies are represented by understanding cell–cell interactions and physiological microenvironments, and by the choice of biomaterials [53,56], which have an important impact on viability and proliferation of the printed cells [56], also providing structural and biochemical support to the cellular components [62]. Silk fibroin has emerged as a promising material for bioink due to the unique properties it has received from nature, which has attributed it good spinnability of the protein by silkworms or spiders [52]. Shear thinning behavior, high printability, cytocompatible gelation and mechanical strength are other relevant properties of fibroin exploited by researchers for bioprinting application [63,64,65]. Furthermore, as bioink, this protein polymer can be physically crosslinked by means of hydrophobic interactions to stabilize the materials without additional chemical reactions or additives [66]. Natural fibers, especially silk, are also appealing materials for bioinspired spinning methods for many biomedical applications [67]. Electrospinning, or electrostatic spinning, consists of providing an electrical field to obtain polymer filaments by a polymer solution, properly controlling morphology and parameters [68] and, for silk spinning, it consists of obtaining silk filaments continuously by means of a fluid forced through a spinneret, by properly monitoring physicochemical parameters, geometry and the shear forces involved [67]. Although some challenges are still related to the use of toxic solvents and pollution, during the past ten years electrospinning techniques have attracted great attention for the development of bioinspired materials [69]. Electrospun nanofibers of natural protein polymers have been proposed to replace the complex cellular environment provided by the extracellular matrix [70], and to produce scaffolds with similar ECM architecture in order to enhance cell adhesion, proliferation, migration and the formation of new tissues [71]. An example of the interesting structure obtained through the electrospinning technique is reported in Figure 2, where both a sample of electrospun fibroin (left) and the corresponding scanning electron microscopy (SEM) analysis (right) are shown. Bio-inspired 3D matrices for mimicking extracellular matrices have also been proposed through other approaches, including additive manufacturing and microscale organ-on-a-chip technologies [72].

## 4. Recent Advances on the Development of Silk Fibroin-Based Wound Dressings

**Figure 3:**
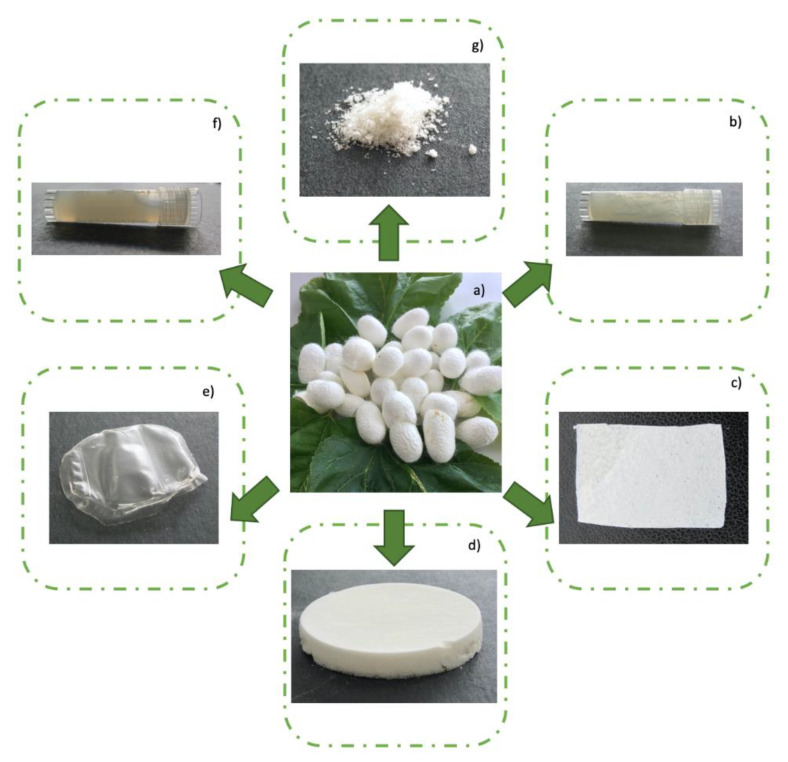
Examples of devices obtained by processing silkworm cocoons (**a**) for wound healing application: fibroin hydrogel (**b**), electrospun fibroin (**c**), sponge (**d**), film (**e**), solution (**f**) and powder (**g**).

The important biological features of silk fibroin from *Bombyx mori* have marked this protein as a good biomaterial for tissue repair and regeneration [73]. Many studies have been performed by several research groups aiming at exploring the great potential of silk fibroin, alone or in combination with other materials and through different processing methods, in order to define advanced approaches for wound healing and tissue engineering applications. Silk fibroin hydrogels, sponges, films, nanofibers etc., have been proposed as wound dressing biomaterials for maintenance of moist environments and gas permeability, and for improved cell response in the different phases of the wound healing process [37,38,39,40]. Figure 3 shows some examples of products developed by the authors Pollini and Paladini, such as fibroin hydrogel (Figure 3b), electrospun fibroin (Figure 3c), sponge (Figure 3d), film (Figure 3e), solution (Figure 3f) and powder (Figure 3g) obtained from silkworms’ cocoons (Figure 3a), which can be exploited for different bioengineering fields and, more interestingly, for wound healing applications.

Among the wound healing application of fibroin, flexible fibroin-based devices for wound dressings, facial masks, contact lenses etc., were also proposed by Bie et al., who developed gel-like fibroin films through direct solubilization of the fibroin fibers in a formic acid/CaCl_2_ solvent, followed by casting on substrates, drying, immersion in water and lyophilization. By this method, the authors obtained controllable hydrophilicity and porosity, along with a favorable environment for cell growth, which suggested good potential for biomedical application [74].

Wang et al. prepared a silk fibroin hydrogel with a dual network structure through a physical and chemical crosslinking with polyacrylamide. The authors aimed at obtaining a stretchable and adhesive material for improved compliance with skin deformation, along with self-healing properties for wound dressing application [75]. Nanofeatured silk membranes were prepared by Karahaliloğlu et al. by the drying of a fibroin solution and then modified through NaOH treatment for dermal wound healing. In particular, the authors aimed at enhancing the functions of fibroblasts and keratinocytes and assessed that their surface modification determined changes in topography, hydrophilicity and chemistry, which improved cell adhesion and proliferation, obtaining cell density on the treated membranes two times higher than on untreated ones [76]. Silk fibroin nanomatrices with large pores were fabricated by Ju et al. by the electrospinning technique combined with porogens. The effect was evaluated on burn wound healing on a rat model, investigating the healing mechanisms by histological analysis and a real time reverse transcription polymerase chain reaction (RT-PCR) assay in comparison with clinically used commercial dressings. The authors demonstrated accelerated re-epithelialization and wound closure in presence of the fibroin nanomatrix, also analyzing the expression patterns of burn-induced cytokines and growth factors associated to wound healing process. In their study, after 28 days, the residual wound area decreased to 4% in the case of fibroin device, while a reduction to 8% and 18% was achieved in case of the tested commercial wound dressings [77].

Silk fibroin scaffolds with water-insoluble amorphous structures were developed by Fan et al. by the lyophilization process. The scaffolds exhibited in vitro improved cell proliferation and neovascularization and were addressed as a promising material for application in soft tissue regeneration [78]. Zhang et al. provided a large in vivo study on the effect of silk fibroin films on full thickness skin defects. Rabbit and porcine models were used for short-term and long-term evaluation, through a macroscopic evaluation of the wound size, the histological analyses, and the calculation of wound closure at different time points. On rabbits, the authors demonstrated the capability of the fibroin devices in reducing the average wound healing time, which was further confirmed in the porcine model. Finally, a randomized single-blind clinical trial on 71 patients demonstrated the successful effect of silk fibroin films in the reduction of both wound healing time and adverse events in comparison with commercial dressings. Of the patients treated with fibroin, 100% healed by 14 days, whereas 88.6% of those treated with the control wound dressings healed by 19 days. The authors concluded that clinical application of silk fibroin films can represent a valuable option for repair and regeneration, with a chance of 72% of early healing [38].

In combination with other materials, as additive or composite materials, fibroin has been extensively studied for improving both chemical–physical and biological properties. For example, Panico et al. developed flexible fibroin films by adding glucose as a plasticizer into silk fibroin. The glucose/fibroin blend was characterized in terms of absorption, mechanical properties, wettability, bacterial biofilm formation, biodegradation and cellular response. The effectiveness of glucose modified silk fibroin films in promoting wound closure was successfully demonstrated in vitro through the scratch assay, which consisted of evaluating cell migration in a wound generated onto a cell monolayer. The migration rate increased to 84% and 100% in the presence of fibroin and a fibroin–glucose blend, thus demonstrating both the biocompatibility and regenerative properties of the device [40]. Wang et al. used the solvent-casting technique to develop fibroin films modified by genipin and glycerol to obtain favorable mechanical properties for wound dressing application. In particular, the authors tested solubility, deformability, breaking elongation and the Young’s modulus of their samples along with the biological properties, suggesting the modified fibroin films as a good candidate for wound care and tissue engineering [79]. A protein-based composite material was developed by You et al., combining egg white and silk fibroin. Both these proteins are biocompatible and biodegradable, and their combination at various ratios was studied by the authors to obtain controlled mechanical properties and enhanced cell response [80]. A bioactive film based on the combination of fibroin with the β-glucan Paramylon was proposed by Arthe et al., which aimed at exploiting the biological activity of fibroin with those associated to Paramylon in terms of enhanced immune response, in order to improve chronic wound healing. The films showed high thermal stability and stiffness, along with improved water absorption and cell proliferation [81].

Tunable mechanical properties and good biocompatibility, water absorption and similar compressive modulus to native skin were obtained by Feng et al., who developed composite protein/polysaccharide sponges through physical crosslinking of silk fibroin and konjac glucomannan [82]. In their study, Li et al. developed a fibroin sponge loaded with insulin-encapsulated silk fibroin microparticles, obtained through coaxial electrospraying of aqueous silk fibroin solution, as a bioactive device for the treatment of chronic wounds. The effect of the biomaterial was evaluated in vivo on diabetic Sprague–Dawley rats, where the results indicated accelerated wound closure, collagen deposition and vascularization. The authors hypothesized improved cell migration and microvascular reconstruction due to the fibroin dressing containing microparticles, along with improved insulin bioactivity due to its sustained release from the microparticles [83]. The effect of fibroin-gelatin microparticles with sizes ranging between 100 μ and 250 μ were developed by Arkhipova et al., who analyzed the wound healing rate in mouse full-thickness skin wounds. The particles injected into the defect area produced accelerated wound healing, improved re-epithelialization and formation of connective tissue, also replacing the damaged derma and stimulating regeneration of subcutaneous muscle and skin appendages. After 21 days, while only 50% control animals healed, all the experimental animals completely healed without cicatrices [84].

A biomimetic scaffold was prepared by Wang et al. through freeze drying combining silk fibroin and sodium alginate, in order to mimic the extracellular matrix and to support tissue regeneration by promoting cell adhesion and proliferation, through a favorable porous structure and good swelling capability [85]. Dorishetty et al. presented biomimetic silk fibroin/cellulose hydrogels for a wide range of tissue engineering applications, including cartilage and meniscus, due to the achieved mechanical properties. For this purpose, the authors investigated the effect of different types of nanocellulose, such as bacterial nanocellulose and cellulose nanofibers, on morphology, structure and performances of the composite hydrogels [86].

Silk fibroin protein was also modified with an acellular goat-dermal matrix to produce a hybrid skin-graft for enhanced wound healing. In vitro studies on murine fibroblasts demonstrated excellent cell viability, proliferation rate and adhesion in the produced scaffold. Moreover, pre-clinical studies on albino mice showed complete wound healing within 14 days, and skin regeneration of full thickness skin without significant inflammatory responses [87].

In combination with elastin, silk fibroin scaffolds were produced by Vasconcelos et al. for mimicking the extracellular matrix in the treatment of burn wounds. Porous scaffolds were obtained by lyophilization, further crosslinked with genipin, thus obtaining scaffolds with different pore sizes and morphologies in relation with elastin ratio and genipin crosslinking. The fibroin/elastin scaffolds supported human fibroblast growth and demonstrated accelerated re-epithelialization and wound closure [88]. Bilayer membranes based on silk fibroin nanofibers and decellularized human amniotic membranes were proposed by Gholipourmalekabadi et al. to overcome the limitation associated to the decellularized human amniotic membrane in the treatment of burns, mainly related to unsatisfactory biodegradation rate, mechanical properties and angiogenesis. The presence of electrospun silk fibroin in the device improved these features and was effective in increasing the angiogenic factors, thus indicating this scaffold as a valuable option for skin tissue engineering [89]. Miguel et al. also aimed at producing a layered structure to mimic both the dermis and epidermis and used the electrospinning technique to produce asymmetric membranes. In particular, silk fibroin and poly (caprolactone) were adopted for the top layer, while the bottom layer was prepared with fibroin and hyaluronic acid loaded with thymol as a herbal drug. The results demonstrated suitable features for wound healing application, in terms of biocompatibility, wettability and mechanical properties [90].

In order to improve cell adhesion and wound healing in skin tissue repair, Wang et al. modified silk fibroin films obtained from a wild silkworm through a polydopamine coating. This resulted in increased roughness and hydrophilicity, which in turn improved absorption properties, adhesion and migration of mesenchymal stem cells in vitro. The histological analyses also indicated promoted epithelization and collagen deposition, without inflammatory effects [91]. The effect of polydopamine coatings was also studied on electrospun silk fibroin membranes by Zhang et al., who found in vitro improved hydrophilicity and fibroblast adhesion and proliferation. In vivo, the authors demonstrated accelerated wound healing in a rat model and stimulated re-epithelialization [92]. Regarding hydrophobicity/hydrophilicity, Keirouz et al. developed silk fibroin composite fibers by blending the silk protein with poly (caprolactone) and poly (glycerolsebacate). Aiming at tunable wettability, the authors obtained a composite biomaterial with good cell response in vitro in terms of fibroblast adhesion and growth, which suggested potential for skin tissue engineering applications [93].

Moreover, a range of bioactive agents such as growth factors, antibiotics or silver nanoparticles have been added to silk fibroin for skin tissue engineering to promote burn and wound healing [71].

## 5. Antibacterial Silk Fibroin

Thanks to the mechanical resistance of its structure, the silk cocoon protects the pupal growth from parasites and predators [94,95], and from biotic and abiotic hazards during the silkworm lifecycle [96]. Moreover, some studies have investigated the role of cocoon components in providing the pupae with protection against bacterial and fungal infections [96,97] and have suggested the presence of effector proteins in silkworm hemolymph with the capability to target and kill bacteria and fungi [96]. Insects do not have an immune system based on antigen–antibody reactions, so they can develop self-defense mechanisms against bacterial infection, inducing for example antibacterial proteins upon bacterial infection [98]. Vaishna et al. showed that seroins, small silk proteins of the domesticated *Bombyx mori*, had antiviral properties against a baculovirus pathogen and inhibited bacterial growth [99]. Even if still under discussion and not fully elucidated yet, sericin has been studied for its antimicrobial effect, these studies mainly addressed to ionic interactions between the protonated amino groups and the negatively charged surface of the bacteria [95,96,97]. The protective functions of the silk cocoons have similarities with the protection provided by the skin to the human body, thus suggesting that the entire cocoon structure, including both fibroin and sericin, can have beneficial effects for wound repair [44,94,95]. Tissue repair can be impaired by many local and systemic factors that can affect one or more phases of the wound healing process [100]. Among them, an increasing interest has been addressed to bacterial colonization and wound infections, which in turn depend on multiples parameters such as the bacterial count, number and types of the strains, response of the immune system etc. [101,102]. Indeed, from a microbiological point of view, the primary function of the skin is to protect the underlying tissues from colonization and invasion by pathogens. [102]. The loss of skin integrity along with the warm and moist wound environment provide favorable conditions for microbial colonization and growth [103]. Moreover, due to the frequent polymicrobial features of wound colonization, frequently involving also pathogenic microorganisms, any wound can become infected [102], thus causing a delay in wound healing, pain and more serious complications [104]. Furthermore, within chronic wounds, bacteria produce biofilm, which contributes to the development of bacterial resistance to antibiotics [101]. When infections occur and the wound healing is impaired, the wound management practices become more complicated and expensive [102]. Recently, wound dressings loaded with antimicrobial agents have emerged as a promising option to reduce the risk of infections, in order to improve the healing process [104,105]. Recent scientific works have explored the potential of different antimicrobial agents, also involving nanotechnological approaches, in combination with bioinspired materials such as fibroin for simultaneous wound healing and their antibacterial properties. In recent years, SF has been also functionalized to obtain a fluorescent material through genetic manipulation or dye feeding methods, for application in drug delivery, bio-imaging, sensing and for monitoring wound healing [106]. In order to obtain synergistic wound healing and antimicrobial properties, some authors have described the potential of fibroin based wound dressing biomaterials, modified through binding or blending with antibacterial agents. For example, composite polyethylenimine (PEI)/silk fibroin bionanotextiles were obtained by Calamak et al. by electrospinning for antibacterial wound dressings [107], while Chan et al. developed a nonwoven mat based on the combination of silk fibroin and a Chinese herbal extract with antibacterial and anti-inflammatory performance [108]. Cai et al. described the fabrication of chitosan/silk fibroin composite nanofibers by electrospinning for wound dressings, demonstrating improved cell adhesion and proliferation and an antibacterial effect against *Escherichia coli (E. coli)* [109]. The combination chitosan/silk fibroin was also proposed by Han et al. for the development of a multi-functional skin substitute. In particular, the authors fabricated a mussel inspired chitosan/fibroin cryogel functionalized by near-infrared light-responsive polydopamine nanoparticles, which exhibited photothermally assisted antibacterial activity [110]. Silk fibroin/graphene oxide (GO) nanofibers were developed by Wang et al. through electrospinning, in order to fabricate an advanced material by exploiting the feature of GO associated to its high number of functional groups and large surface-to-volume ratio. The authors demonstrated the antibacterial capability of the material on *E. coli* in comparison with pristine SF, and applied this effect to the capability of GO to destroy the bacterial membranes and to lead an efflux of intracellular substances [111]. Silver compounds have also been proposed by some authors to mimic the skin tissue in the treatment of skin trauma or burns, such as porous silk fibroin sponges produced by freeze drying and treated with silver sulphadiazine proposed by Çakır et al., which resulted in inhibited bacterial growth [112].

**Figure 4:**
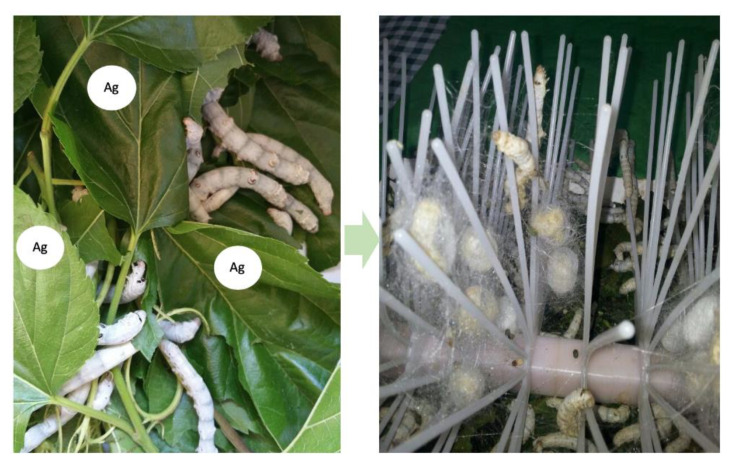
Representative pictures describing a method for obtaining intrinsically antibacterial silk fibroin. Silkworms fed on silver modified diet (**left**) produce silver doped cocoons (**right**) which can be processed to develop products with antibacterial properties.

Promising combinations of silk fibroin and silver nanoparticles were suggested by some authors aiming at developing wound dressings for preventing wound infection and simultaneously promoting wound healing [113]. Pei et al. proposed sponges based on a silk fibroin/carboxymethylchitosan composite doped with silver nanoparticles and demonstrated their antibacterial activity against *Staphylococcus aureus* (*S. aureus*) and *Pseudomonas aeruginosa* (*P. aeruginosa*), along with improved water absorption and water transmission rate [113]. Calamaka et al. produced silver/fibroin composite nanofibers and investigated the effect of the fibroin structure (random coil or beta sheet) on the release of silver ions and on antibacterial capability on *Staphylococcus aureus* and *Pseudomonas aeruginosa* [114]. Silk fibers functionalized with silver nanocolloids were proposed by Dhas et al., who demonstrated that the incorporation of silver nanoparticles (AgNPs) produced an enhancement of thermal and mechanical properties and antibacterial capability against *P. aeruginosa and S. aureus* without a toxic effect on fibroblasts [115]. An innovative method was developed and patented by the authors Pollini and Paladini to obtain intrinsically antibacterial silk fibers directly from *Bombyx mori* silkworms [116]. The authors found that silver-doped silk proteins can be obtained by feeding silkworms with a silver-modified diet (Figure 4).

This method, which does not affect the lifecycle of the silkworms and does not involve any additional chemical–physical treatment, allows the production of silk proteins products with simultaneous regenerative and antibacterial properties for wound healing application [116].

Along with the well-known antimicrobial properties [117,118,119,120], some studies have also demonstrated a role of silver nanoparticles in wound healing, which can further improve the biological properties of fibroin through the development of fibroblasts into myofibroblasts, thus promoting wound contraction and healing rate, and stimulating keratinocyte proliferation [121,122,123,124].

In combining silver with fibroin, this silk protein also provides multiple functions which can be exploited from a technological point of view. Due to the presence of tyrosine amino acid residues, silk fibroin has strong electron donating properties that can reduce Ag^+^ to Ag [113,125]. In their reaction system, Fei et al. exploited the reducing properties of fibroin to produce a silk fibroin–silver nanoparticle composite via an environmental-friendly process and demonstrated antibacterial activity against methicillin-resistant *Staphylcoccus aureus* and biofilm [125]. Based on the fibroin capability to reduce silver ions, Babu et al. have proposed silver oxide nanoparticles embedded in silk fibroin spuns for synergistic antibacterial and wound healing properties, applying the novelty of their work to the simultaneous formation and adhesion of Ag_2_O nanoparticles to the surface of the reducing agent [126]. Raho et al. synthesized composite hydrogels made of regenerated silk fibroin stabilized with CarboxymethylCellulose-Na and loaded with different amounts of silver nanoparticles, suggesting this novel material as part of a wound dressing with regenerative and antimicrobial properties tested against *E. coli*, *S. aureus S. epidermidis*, *Methicillin Resistant Staphylococcus aureus* (*MRSA*), *P. aeruginosa*, *C. albicans and Fluconazole-resistant Candida albicans* (*FRCA*) [25].

Different contents of silver nanoparticles were incorporated and tested also by Mehrabani et al., who developed silver/silk fibroin/chitin nanocomposite scaffolds by freeze drying for the treatment of wound infections. Good cytocompatibility, biodegradation, mechanical properties and antimicrobial activity against *E. coli, S. aureus* and *Candida albicans* (*C. albicans*) were demonstrated by the authors, who suggested the wound dressing material as a promising option for in vivo uses [127]. Among various available options, silk has demonstrated great potential in a wide range of medical/pharmaceutical applications, thus contributing to the development of novel approaches in tissue engineering and fabrication technologies [9,128].

## 6. Conclusions

Nature has originated a huge number of biomaterials with high levels of sophisticated structures and functions, evolved over many thousands of years in different environments. The term “bioinspiration” refers to a product or process which can translate a certain biological design into useful technologies, such as self-cleaning surfaces, self-healing materials, natural interfaces etc. [9]. Many efforts have been made in the last decade in the development of biomimetic materials with similarity to the natural materials of the body [96] and bioinspired engineering has been put forward as a valuable tool for the development of clinically relevant materials and structures for regenerative sciences [9]. For engineers and clinicians, a great challenge in repair/regeneration approaches is represented by the necessity to closely mimic the complex architectures of the human body and the properties and functions of the ECM of the native tissues [129]. For this purpose, material engineering inspired by the wide range of adaptions in nature represents a useful tool for designing novel clinically relevant materials and structures for regenerative medicine [9]. Bioinspired research will continue to focus on the design of functional biomaterials to control cell–matrix interaction at any length scale [10]; however, some challenging aspects still require more investigation on bioinspired and biomimetic systems, mostly related to the comprehensive understanding of the structure–property relationships of the biological world, the translation of its motifs to a wide combination of materials [2,8], and the regenerative and immunological processes [10]. Among tissue engineering applications, wound management still represents a huge challenge for clinicians, and is also a big commercial enterprise, involving a market of about 15 billion US dollars [130]. It continuously requires novel systems and devices to improve clinical outcomes and to provide more effective therapeutic options because of the multiple factors involved in the healing process, which can adversely affect the different stages of the wound healing and determine the failure of conventional approaches [106]. An ideal wound dressing should be able to maintain a moist environment while removing the excess exudate, should protect the wound from contaminants and from further trauma also when removed, and should ensure comfort and good thermal conditions and gaseous exchange [131].

In this scenario, proteins in general could be employed in addition or in place of classical synthetic polymers [2] and silk materials, particularly, have attracted more attention because of their excellent bioresponse and capability to be replaced by native tissues [1].

Compared with other synthetic or natural polymers for biomedical application, silk fibroin presents several advantages. Among them, the thermal stability up to about 200 °C and environmental stability are of great importance for biomedical application [132]. Indeed, compared to other fibrous proteins such as collagen, fibroin offers multiple options for sterilization, such as ethylene oxide, γ-radiation and 70% ethanol. Autoclaving of fibroin scaffolds does not affect their structure and properties, while collagen denatures at these temperatures [133,134]. Moreover, compared to biodegradable polymers such as poly(lactic-co-glycolic acid) (PLGA) and poly(lactic acid) (PLA), which can increase local pH and affect cellular processes due to the degradation products of aliphatic polyester, the protein biopolymers have degradation products mostly consisting of amino acids that can be resorbed by cells [134]. Moreover, the degradation of silk can be controlled in function of processing parameters and crystallinity [132]. In terms of biological responses involved in wound healing application, SF biomaterials demonstrated higher activity compared to commercially available collagen materials. Hashimoto et al. demonstrated different behaviors in human fibroblasts cultured on collagen biomaterials and fibroin-based biomaterials. In particular, silk fibroin induced higher gene expression for wound repair than collagen film, and also higher cell motility due to weaker cell–fibroin interactions than collagen [135]. Due to its intrinsic biological features involving improved cell migration and proliferation, and wound healing properties, silk fibroin represents an extremely valuable option among biomaterials [39] as a good candidate for fabrication of novel natural wound dressings for a wide range of skin injuries. Some limitations still need to be solved for a systematic use of silk proteins in biomedical fields, among which is the limited number of companies producing high quality non-hydrolyzed fibroin.

Furthermore, compared to other large-scale produced polymers, fibroin is more expensive and involves sericulture and silkworm rearing, which represent an intense part of the activities for silk production [136]. However, beyond its use in the textile field, the development of multi-level silk fibroin structures can become the focus of future research in connection between academic and industrial sectors [48], offering multiple opportunities for future healthcare applications [132].

## Abbreviations

ECM	Extracellular matrix
FDA	Food and Drug Administration
Gly	glycine
Ala	alanine
SF	silk fibroin
SEM	scanning electron microscopy
RT-PCR	reverse transcription polymerase chain reaction
PEI	polyethylenimine
*E. coli*	*Escherichia coli*
GO	graphene oxide
*S. aureus*	*Staphylococcus aureus*
*P. aeruginosa*	*Pseudomonas aeruginosa*
AgNPs	silver nanoparticles
*S. epidermidis*	*Staphylococcus epidermidis*
*MRSA*	*Methicillin Resistant Staphylococcus aureus*
*C. albicans*	*Candida albicans*
*FRCA*	*Fluconazole-resistant Candida albicans*
*PLGA*	*poly(lactic-co-glycolic acid)*
*PLA*	*poly(lactic acid)*
